# Biomaterials Based on Chitosan and Polyvinyl Alcohol as a Drug Delivery System with Wound-Healing Effects

**DOI:** 10.3390/gels9020122

**Published:** 2023-02-01

**Authors:** Simona Petronela Gherman, Gabriela Biliuță, Adrian Bele, Alina Mirela Ipate, Raluca Ioana Baron, Lăcrămioara Ochiuz, Adrian Florin Șpac, Daniela Elena Zavastin

**Affiliations:** 1 Faculty of Pharmacy, “Grigore T. Popa” University of Medicine and Pharmacy Iasi, 16th University Str., 700115 Iasi, Romania; 2 “Petru Poni” Institute of Macromolecular Chemistry of Romanian Academy, 41 A, Grigore Ghica Voda Alley, 700487 Iasi, Romania

**Keywords:** L-arginine, caffeine, wound healing, entrapment efficiency, sorption isotherm, drug release, permeation kinetics

## Abstract

The excellent biological properties of chitosan (CS) together with the increased oxygen permeability of polyvinyl alcohol (PVA) were the prerequisites for the creation of a wound healing dressing that would also function as a system for L-arginine (L-arg) and caffeine (Caff) delivery. Using the freezing/thawing method, 12 hydrogels were obtained in PVA:CS polymer ratios of 90:10, 75:25, and 60:40, and all were loaded with L-arg, Caff, and the mixture of L-arg and Caff, respectively. Afterwards, an inorganic material (zeolite–Z) was added to the best polymeric ratio (75:25) and loaded with active substances. The interactions between the constituents of the hydrogels were analyzed by FTIR spectroscopy, the uniformity of the network was highlighted by the SEM technique, and the dynamic water vapor sorption capacity was evaluated. In the presence of the inorganic material, the release profile of the active substances is delayed, and in vitro permeation kinetics proves that the equilibrium state is not reached even after four hours. The synergy of the constituents in the polymer network recommends that they be used in medical applications, such as wound healing dressings.

## 1. Introduction

Skin is considered the largest organ and acts as a barrier against a number of physiological and functional factors, thus preventing the invasion of microorganisms [1,2]. Normally, skin tissue can regenerate, but deep and/or extensive tissue trauma can result in a number of major complications, such as fungal and bacterial infections [3]. For the care of wounds, and also for the regeneration and proliferation of tissues over time, the development of new products containing biocompatible and desirable materials was considered [4]. The flexibility of hydrogels allows them to be used as dressings for wound care, and their structure allows the maintenance of optimal wound moisture, determines the absorption of exudate, does not adhere to the wound, and is non-invasive for the patient [4,5]. The three-dimensional structure of the polymer network in hydrogels facilitates the loading and release of active compounds at the target site, and direct application to the skin causes the passage of the medicinal substance through the tissue and thus avoids the first hepatic passage [5,6]. By maintaining optimal moisture in the wound and with the delayed release of the active pharmaceutical ingredient, the frequency of dressing replacement is reduced, so that hydrogels can be included in the category of “smart” materials [7,8]. The performances of hydrogels are established from the synthesis phase, and they depend not only on the nature of the polymers, but also on the method used to obtain them [9]. The most common hydrogels in the biomedical and pharmaceutical fields are based on polyvinyl alcohol (PVA) and chitosan (CS) [5,9,10,11,12,13,14,15].

Polyvinyl alcohol (PVA) is a biodegradable semi-crystalline synthetic polymer that has been used in biotechnology, such as tissue regeneration, wound dressings, and drug delivery systems. PVA-based dressings have excellent properties, such as their biodegradability, biocompatibility, lack of toxicity and low cost. PVA contains a secondary alcohol group attached to a linear carbon chain and, depending on the degree of hydrolysis, presents different chemical properties and different degrees of solubility and crystallinity, which form very flexible films with increased permeability for oxygen and water vapor, which increases the rate of wound healing [9,16,17,18]. PVA is easily mixed with other polymers such as chitosan, a natural polysaccharide that has excellent properties such as biodegradability, biocompatibility, non-toxicity, and antimicrobial properties, and it exhibits a hemostatic and regenerative effect in tissue engineering [4,5,19,20]. Chitosan is one of the most important biopolymers with applications in the industrial field (removal of contaminants from wastewater), food (food additives, packaging, and preservatives), agriculture (coatings for seeds and fertilizers, and controlled agrochemical discharge), paper manufacturing, cosmetic products, tissue regeneration, wound healing, and drug transport [21,22,23,24,25,26,27]. The three-dimensional structure of CS-based hydrogels allows the loading and release of the drug through release techniques sensitive to pH and temperature [28,29]. The antibacterial properties of CS are determined by its positive charge that causes interactions with the negative charges of proteins, anionic polysaccharides, and nucleic acids in the bacterial membrane [20]. Another remarkable property of CS is its interaction with mucus and epithelial cells resulting in increased epithelial permeability [20].

The method of obtaining hydrogels is very important, especially in biomedical and pharmaceutical applications, where the presence of residues of crosslinking agents and solvents would increase the toxicity and unwanted effects of the pharmaceutical active ingredient [9]. By using the freeze–thaw process through successive cycles, non-toxic hydrogels (cryogels) are obtained, which do not require crosslinking agents, so that the purification step is removed by their absence. Through this method, the polymer chains exhibit much stronger interactions leading to a stable hydrogel structure and tunable mechanical properties [9,30].

The potential of such wound dressings increases considerably by adding active ingredients. Caffeine is a non-selective antagonist of adenosine receptors, which through increased angiogenesis induces wound healing [31]. Caffeine, through its antioxidant properties, has the role of neutralizing free radicals that form on tissue lesions and, thus, cell proliferation takes place in order to accelerate wound healing [32,33]. Research on arginine (L-arg) has highlighted its bioactive role, which is due to its excellent biosafety and antimicrobial properties, so arginine therapy may be a promising strategy for wound healing [34,35]. It was reported that nitric oxygen (NO) derived from L-arg has great advantages for accelerating wound healing, by enhancing angiogenesis and reducing bacterial infections [36,37].

This study aimed to develop new dressings based on non-toxic and biodegradable materials with antimicrobial action to be used in wound care therapy.

## 2. Results and Discussion

### 2.1. FTIR Spectroscopy

**Figure 1:**
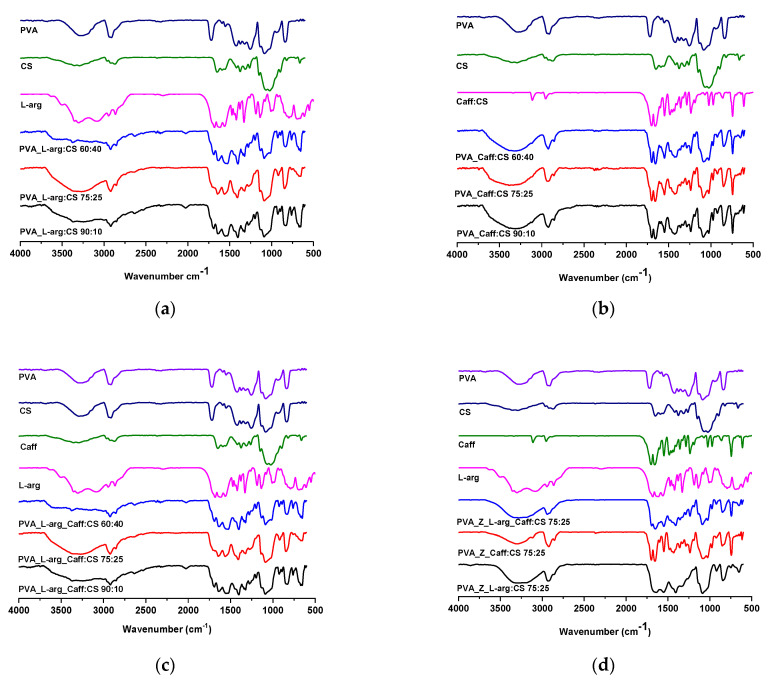
FT-IR spectra of hydrogels (**a**) loaded with L-arginine; (**b**) loaded with caffeine; (**c**) loaded with L-arginine and caffeine mixture, and (**d**) containing zeolite.

Figure 1a shows the FT-IR spectra of the three hydrogels based on PVA and CS, loaded with L-arg. L-arg presents a characteristic signal at 3082 cm^−1^ which is due to –NH stretching, and at 1681 cm^−1^ which corresponds to –NH_2_ bending. The stretching movement of the C=O group appears in the spectrum at 1560 cm^−1^, and the bond due to the O–H group is observed at 1500 cm^−1^ [38].

In the hydrogels, the characteristic signals of chitosan are found at 1649 cm^−1^, being specific to amide I, while the one at 1560 cm^−1^ is attributed to amide II [39]. The main bands of PVA are observed at 3292, 2912, 1718, 1421, 1255, 1085 and 839 cm^−1^ and are assigned to the O–H stretching vibration of the hydroxyl group, the CH_2_ asymmetric stretching vibration, the stretching of the carbonyl group C=O, the C–H bending vibration of the CH_2_ group, the C–H bending vibration, the C–O stretching of acetyl groups, and the C–C stretching vibration, respectively [40,41].

Additionally, the spectra of PVA–CS hydrogels show a peak at 1276 cm^−1^ which is characteristic of C–N stretching. From the FT-IR spectra of PVA:CS hydrogels in different ratios, it can be seen, as expected, that the signals of amide I (1645 cm^−1^) and II (1560 cm^−1^) groups increases with the increase in the chitosan ratio and the signals of the C–H bending groups of the CH_2_ group in PVA that initially appear at 1421 cm^−1^ decrease with the decrease in PVA concentration.

For all three hydrogels loaded with L-arg in different polymer ratios, it was found that the specific signals of the OH group in PVA from 3292 cm^−1^ were shifted to a lower wavenumber, up to 3200 cm^−1^, which suggest the formation of additional hydrogen bonds between PVA and L-arginine [42].

From the FT-IR spectra of the hydrogels loaded with caffeine, which are shown in Figure 1b, a broad peak around 3300 cm^−1^ can be observed which is attributed to the N–H stretching vibration; the C–H stretching signal from the aromatic structure appears at 3106 cm^−1^ and at 2956 cm^−1^, and the peak around 1700 cm^−1^ is due to the aromatic ring –C=N [43]. With increasing CS concentration in the hydrogels, there is an increase in the intensity of the peak at 1655 cm^−1^ for amide I and the peak at 1560 cm^−1^ for amide II. The decrease in the intensity of the peak at 3290 cm^−1^, which is specific to the OH group in the polymer, is correlated with the decrease in PVA concentration in the obtained hydrogels.

Figure 1c shows the FT-IR spectra of the hydrogels loaded with the mixture of the two drugs, so the signal around 1700 cm^−1^ is specific to the aromatic ring –C=N in the Caff structure, and the one at 3082 cm^−1^ is due to the stretching –NH from the L-arg structure. Peaks unmodified intensity, both for Caff and L-arg, indicates that the drugs are found in hydrogels. The decrease in the intensity of the peak at 3290 cm^−1^, which is specific to the OH group in the polymer, is correlated with the decrease in PVA concentration in the obtained hydrogels. With increasing CS concentration in the hydrogels, there is an increase in the intensity of the peak at 1655 cm^−1^ for amide I and the peak at 1560 cm^−1^ for amide II.

The FT-IR spectra for polymer-based hydrogels and zeolite as an inorganic material loaded with L-arginine, caffeine, and the mixture of the two, respectively, suggest that the interaction between the drugs and the other components is mostly related to the polar groups (Figure 1d). In all three spectra, characteristic absorption bands specific to PVA appear, namely, 3200–3300 cm^−1^ is the band specific to intramolecular and intermolecular hydrogen bonding which is due to high hydrophilic forces. In the 2850–3000 cm^−1^ region, typical alkyl C–H absorption broad bands appear (2939 and 2927 cm^−1^). The presence of L-arginine in the samples is evidenced by the absorption bands at 1560 cm^−1^, which are specific to the C=O group in L-arginine (1548 cm^−1^ and 1552 cm^−1^, respectively), while the presence of caffeine in the hydrogel is noted by some characteristic vibrations of caffeine peaks, namely those at 1700 cm^−1^ which are due to the aromatic ring –C=N. From the FT-IR spectra of hydrogels based on PVA_Z:CS loaded with the mixture of the two drugs (L-arginine and caffeine), the specific signals of the two substances, as well as signals characteristic of the zeolite L band at 1080 cm^−1^ (probably Si -O-Si), can be observed [44]. By introducing zeolite L into the hydrogels, the semi-crystalline structure of PVA was not modified.

### 2.2. Morphological Characterization via Scanning Electron Microscopy (SEM)

**Figure 2:**
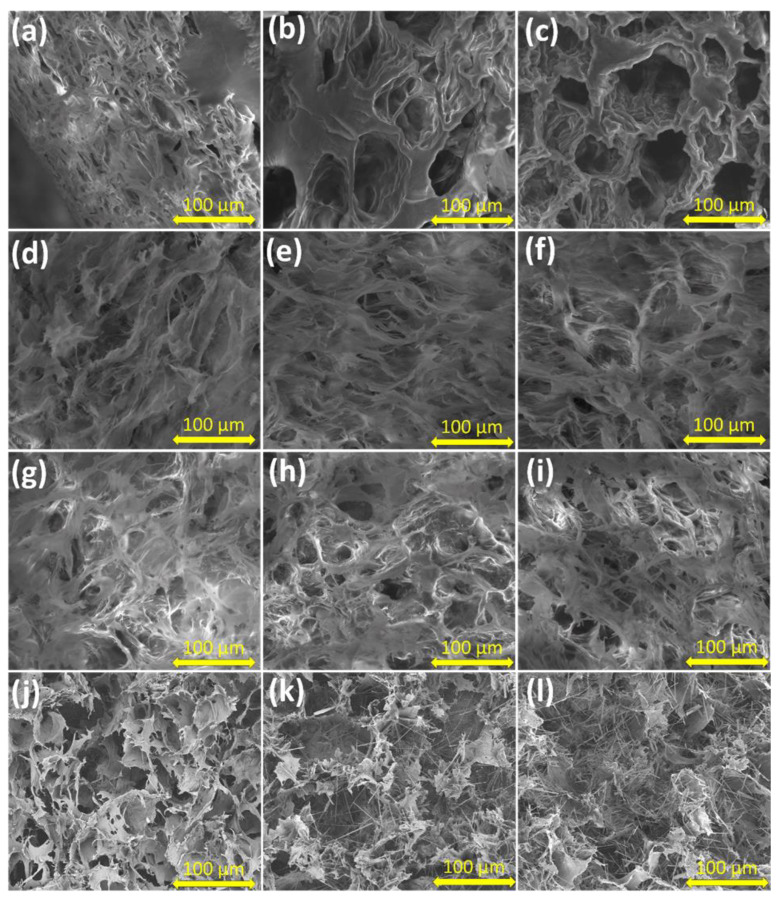
SEM images of hydrogels: (**a**) PVA_L-arg:CS 90:10; (**b**) PVA_L-arg:CS 75:25; (**c**) PVA_L-arg:CS 60:40; (**d**) PVA_Caff:CS 90:10; (**e**) PVA_Caff:CS 75:25; (**f**) PVA_Caff:CS 60:40; (**g**) PVA_L-arg_Caff:CS 90:10; (**h**) PVA_L-arg_Caff:CS 75:25; (**i**) PVA_L-arg_Caff:CS 60:40; (**j**) PVA_Z_L-arg:CS 75:25; (**k**) PVA_Z_Caff:CS 75:25; (**l**) PVA_Z_L-arg_Caff:CS 75:25.

The morphological appearance of the hydrogels was determined by scanning electron microscopy and is shown in Figure 2a–l. It can be observed from the images that all the samples show 3D porous structures with regular interconnected areas. With the increase in the percentage of chitosan, the porosity of the network changes and the size of the pores increases, and this has been proven to facilitate a faster release of the drug from the hydrogel structure [45]. The homogeneous appearance of the pores of the hydrogels can be correlated with the homogeneous loading of the entire PVA_CS polymer network.

By introducing the zeolite L nanoparticles, (Figure 2j–l), an increase in the size of the pores and a decrease in the number of pores can be observed. The lack of agglomeration or phase separations of zeolite L nanoparticles denotes a uniform distribution between the inorganic phase and the PVA_CS_drug system.

### 2.3. Dynamic Water Vapor Sorption Capacity

The dynamic water vapor sorption capacity analysis allows establishing the relative humidity conditions of the environment that influence the stability and quality process of the materials, and at the same time, by tracing the sorption isotherms, the hydrophilic or hydrophobic nature of a material can be established. The sorption isotherms of the prepared hydrogels were recorded in the range of relative humidity (RH) of 0–90% at a temperature of 25 °C.

According to the IUPAC classification, all hydrogels obtained show a Brunauer–Emmett–Teller (BET) multilayer sorption isotherm, type V, specific to mesoporous materials [46,47]. Materials showing such isotherms are hydrophobic. Water molecules are captured by condensation in the free meshes of the capillary network, and only a small part of the water molecules can attach to the surface functional groups [48].

**Figure 3:**
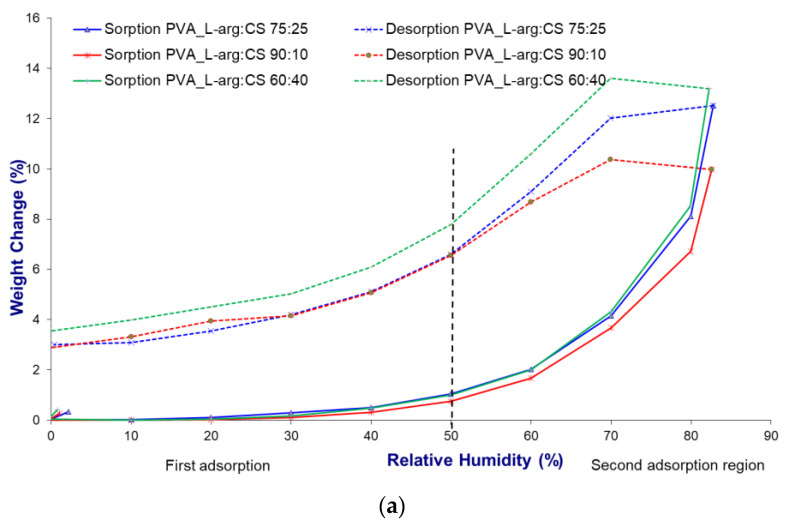

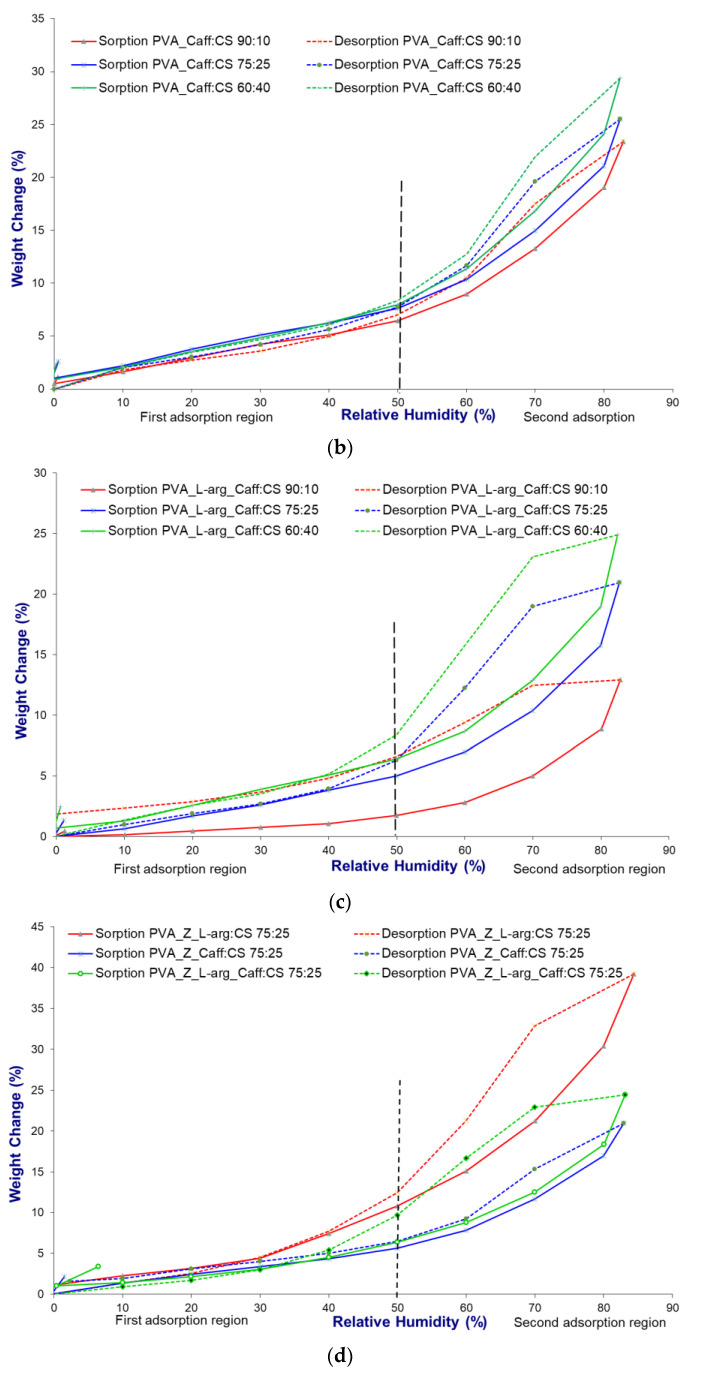
Sorption/desorption isotherms of hydrogels (**a**) loaded with L-arginine, (**b**) loaded with caffeine, (**c**) loaded with L-arginine and caffeine mixture, and (**d**) containing zeolite and polymer ratio PVA:CS 75:25.

The isotherms of all samples (Figure 3a–d) show hysteresis between sorption and desorption over the whole studied humidity range. The dry mass for the two processes shows differences that are due to the condensation and evaporation process that takes place in the pores of the hydrogels. To investigate the sorption process, the isotherms were divided into two regions, one with low relative humidity, up to 50%, and one with high relative humidity, above 50%. In the region with low humidity, it is observed that the isotherm does not increase sharply, which indicates a lack of affinity between the hydrogel constituents and the water molecules. At a humidity above 50%, a sharp increase in sorption capacity was found, which was primarily due to capillary condensation occurring in interconnected capillary channels. Moreover, in the first region, the water vapor pressure is not sufficient to facilitate the penetration of water into the internal structure of the hydrogels. On the other hand, with the increase in humidity, there is an increase in the pressure of the water molecules, which facilitates the penetration into the internal structure of the hydrogels, thus explaining the different sorption capacity in the two regions [49,50]. The sorption isotherms for all hydrogels are similar in shape and are specific to hydrophobic mesoporous adsorbents [47]. 

The hydrophilic character of chitosan, determined by the presence of amino and hydroxyl groups, facilitates the increase in water sorption capacity, and when it is mixed with PVA, electrostatic interactions and hydrogen bonds appear, which leads to a decrease in the number of functional groups [47,49]. The experimental results showed that with the increase in the amount of CS in the hydrogels, there is an increase in the water sorption capacity. At the same time, the type of drug loaded in the hydrogel influenced the amount of adsorbed water, thus it formed an amide bond between the carboxyl group of L-arg and the amino groups of chitosan [50,51]. The formation of such bonds led to the decrease in the water sorption capacity over the entire range of relative humidity, so at 50% humidity, the maximum sorption was 0.53% d.b. (% dry basis), and at 80% humidity, the maximum was 6.56% d.b (Figure 3a). The polar nature of both chitosan and arginine prevents the release of water from inside the crystalline network, so the desorption isotherm is different from the sorption one, and at 50% humidity, the maximum sorption is 10.34% d.b. In this case, the difference between sorption and desorption processes is 9.81% d.b. By combining the hydrophilic nature of chitosan with that of caffeine, given by the four nitrogen atoms that are able to form hydrogen bonds, the water sorption capacity increased (Figure 3b) [52]. Both in the low humidity and high humidity area, the sorption capacity increases steadily; at 50%, it is 6.52% d.b., and after 60% humidity, the increase is sudden, reaching 29.35% d.b. The desorption isotherm of the caffeine-loaded hydrogels overlaps the sorption isotherm, and the % mass of the samples for the two processes show no differences, indicating only the penetration of water into the crystal network without the formation of hydrogen bonds. For the hydrogels loaded with a mixture of L-arg and Caff (Figure 3c), the sorption capacity increases with the increase in the amount of chitosan added in the polymer formula of the hydrogel. The last set of hydrogels in a PVA:CS polymer ratio of 75:25 and zeolite as an inorganic desiccant material loaded with drugs shows increased values of the amount of water adsorbed (Figure 3d). The results confirmed that the introduction of L-arg through the highly polar guanidine group increases the sorption capacity, reaching a value of 22.99% d.b. at 80% humidity. For the same polymer recipe but loaded with Caff, the maximum value, at 80% humidity, is 13.45% d.b., and for the mixture of L-arg and Caff, at 80% humidity, the value is 14.15% d.b. In these hydrogels, the isotherms of the desorption process overlap with those of sorption, which indicates only a physical penetration of water molecules inside the polymer network without the formation of hydrogen bonds.

### 2.4. Estimation of Drug Loading and Entrapment Efficiency

The entrapment efficiency (*EE*) and degree of loading of the drug into the hydrogel were determined by a spectrophotometric method. The wavelength at which L-arginine absorbs in phosphate buffer (pH 7.4) is 208 nm and shows linearity in the concentration range 5–35 µg/mL, with regression coefficient r^2^ = 0.9995. Caffeine absorbs at a wavelength of 273 nm, and the linearity is in the concentration range of 5–25 µg/mL, with the regression coefficient r^2^ = 0.9999. The values of the regression coefficient indicate that the drug release follows Beer’s law within the specific concentration range [53]. The calculations of the drug loading and *EE* were based on the calibration curve. The calibration curve for L-arg is absorbance = 0.0142∙concentration + 0.0465, and for Caff it is absorbance = 0.0498∙concentration = 0.0119.

The results of this study are shown in Table 1 and presented a direct correlation between drug loading and entrapment efficiency. From the L-arg and Caff mixture samples, quantitative analysis of L-arg could not be performed because it absorbs at a shorter wavelength than Caff, so the spectra overlap and does not allow its determination.

The entrapment efficiency varies within very small limits, of 99% for the samples that do not contain zeolite, and up to 85.09% for the samples with inorganic material which are loaded with the L-arginine and caffeine mixture. This decrease can be explained by the occupation of the polymer network with zeolite, which, being inorganic in nature, makes it difficult for the drug molecules to bind to the polymer network sites. The microporous structure of the zeolite occupies the polymer network, which causes a decrease in the percentage of medicinal substance, can both penetrate inside the pores of the zeolite and be dispersed on the surface of the material [54,55].

### 2.5. In Vitro Drug Release and Permeation Studies

**Figure 4:**
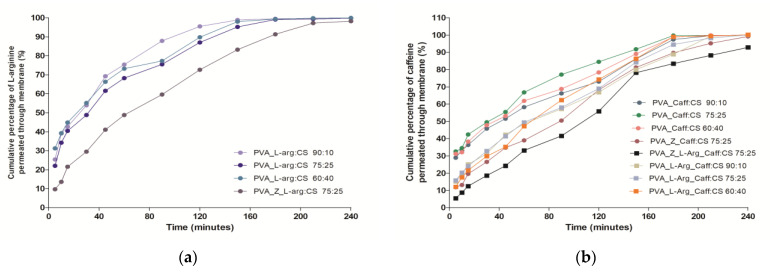
In vitro release profile: (**a**) L-arg and (**b**) Caff from the hydrogel samples.

The results of the drug release from the structure of the hydrogels, shown in Figure 4a,b, are consistent with the FTIR analysis which revealed the presence of weak drug-polymer bonds.

**Figure 5:**
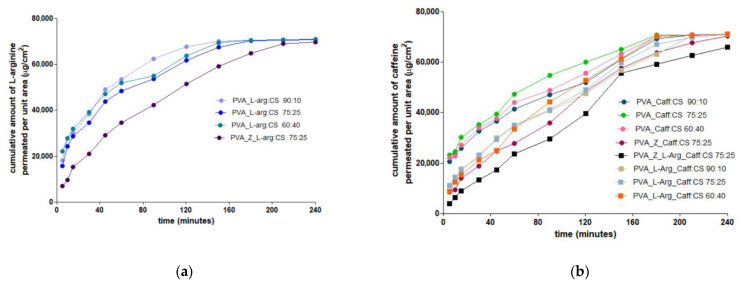
In vitro permeation of (**a**) L-arg and (**b**) Caff from hydrogels across the chicken skin membrane.

The analysis of the release profile for L-arg shows an accentuated release (burst release) of over 40% in the first 15 min regardless of the PVA:CS polymer ratio, and with the addition of zeolite, there is a decrease in the release, reaching 21.55%, result that is due to a slight encapsulation of L-arg in the pores of the inorganic material (Figure 4a). The L-arg permeation through the chicken skin membrane reached the steady state after 2 h for the formula based on PVA and CS, while in the case of PVA_Z_L-arg_CS 75:25, the steady state could not be reached within four hours (Figure 5a).

For Caff, when it is loaded alone in the polymer material, it shows the same enhanced release of more than 40% in the first 15 min, but when it is loaded together with L-arg in the polymer matrix, the release is delayed, reaching a maximum of 25.05% in the first 15 min (Figure 4b). The decrease in Caff release is even more pronounced when zeolite is also present in the polymer matrix, the release percentage being 19.60% in the first 15 min, and after 4 h, the caffeine release reaches a value of 99.2%. After 3 h, all formulas based on polymers exclusively achieved a steady state for the permeation of Caff across the biological membrane (Figure 5b). Once again, the zeolite formulation in the hydrogel matrix determined a prolonged release profile of Caff from hydrogels. The accelerated release profile of arginine satisfies the wound healing modality, when, in the first phase, a large amount of arginine is needed to inhibit infection, promoting angiogenesis and granulation formation, which leads to wound healing with less scars [37]. Hydrogels prepared and loaded with caffeine, which have a prolonged release, can be used on wounds that have reached the second phase, namely the inflammatory phase [56].

### 2.6. Analysis of In Vitro Drugs Release Kinetics

In order to correlate the data obtained from the active substance release studies with the characteristics of the pharmaceutical form, the release profiles of L-arg and Caff from the hydrogels were studied. The data of the in vitro release profile were studied on the four main kinetic models and the results obtained were interpreted by means of the statistical criteria Akaike information criterion (AIC) and correlation coefficient (R^2^). The results of the release profile for L-arg are presented in Table 2, and for Caff in Table 3.

The values of the main statistical parameters indicate that the obtained formulations do not follow an ideal zero-order kinetics, nor a first-order kinetics, which depends on the initial concentration of the drug [57]. The R^2^ values for the Higuchi kinetic model and the Korsmeyer–Peppas kinetic model do not differ significantly, but the lower AIC values from the Korsmeyer–Peppas model indicate for all hydrogels that the release of the active substance occurs through the diffusion phenomenon [58]. At the same time, the diffusion mechanisms are indicated by the values of the diffusion exponent (n) from the Korsmeyer–Peppas equation.

The values of the diffusion exponent, specific to the kinetic equation of Korsmeyer–Peppas (Table 2), indicate for L-arg a release by Fickian diffusion when the drug is loaded alone in the polymer network, regardless of the PVA:CS ratio (n = 0.3–0.35). When the polymer network also contains zeolite, the release of L-arg is achieved through diffusion anomalies, with n = 0.54 (diffusion and erosion of the polymer network) [59,60].

From Table 3 it can be seen that for hydrogels loaded only with caffeine, regardless of the polymer ratio, the release of the drug takes place by Fickian diffusion (n ˂ 0.45) [59,60]. For the hydrogels that are loaded with a mixture of Caff and L-arg, and also for those that have zeolite added in the formula, the values of the diffusion exponent, n, are between 0.54 and 0.78, which indicates a non-Fickian release mechanism, for which the release occurs by diffusion coupled with erosion [59,60].

For the release of both L-arg and Caff, the Korsmeyer–Peppas model proved superior to the zero-order, first-order, and Higuchi kinetic models. The presence of zeolite in the hydrogel structure determines a drug release mechanism through diffusion and erosion processes.

## 3. Conclusions

Wound healing is a complex process that requires the best materials capable of ensuring optimal tissue moisture, functioning as an antibacterial barrier, and being non-invasive for the patient. The synergism given by the increased oxygen permeability of PVA, the antibacterial, antimicrobial and hemostatic effect of CS, and the antimicrobial properties of L-arg together with the antioxidant, anti-inflammatory, and antibacterial properties of Caff were the prerequisites for making the 12 hydrogels by the freeze–thaw method. The obtained hydrogels are in PVA:CS polymeric ratios of 90:10, 75:25, and 60:40 and were loaded with L-arg, Caff, and the mixture of the two, respectively. All hydrogels have a microporous structure, and the size of the pores increases with the increase in the amount of CS. The experimental data showed an increased water vapor absorption capacity with the increase in the amount of CS in the polymer network. The addition of L-arg in the polymer network causes a decrease in the water vapor sorption capacity due to the formation of hydrogen bonds between chitosan and L-arginine. When Caff is added to the hydrogels, there is a sharp increase in the sorption capacity, and by superposition of the desorption isotherm on the sorption isotherm, physical penetration of water vapor into the hydrogels is indicated, without the formation of hydrogen bonds. The loading tests of hydrogels that contain only organic material show a loading efficiency of over 98.8% and those that also contain zeolite as an inorganic material show a slight decrease in loading efficiency with values between 85.09 and 95.88%. Release tests in phosphate buffer indicate an immediate release of both L-arg and Caff when they are in the polymer network, and the presence of zeolite in the hydrogel determines a modified release for both active substances. The zeolite causes a prolonged release effect, both of L-arg and of Caff, and both due to the phenomenon of occupying the pores of the network by the zeolite, but also due to the fact that the zeolite delays the phenomenon of hydration of the polymer network. The release of Caff in the presence of L-arg is a slower phenomenon that is favorable to the healing process considering the sequence of the biological stages of scarring.

## 4. Materials and Methods

### 4.1. Reagents

Chitosan with a low molecular weight (molecular weight of 167,494 Da–determined by the viscosimetric method) [61] and 75% of degree of deacetylation, polyvinyl alcohol (average molecular weight of 85,000–124,000 Da and a hydrolysis degree of 99%), caffeine, and L-arginine were purchased from Sigma-Aldrich (St. Louis, MI, USA). All other chemicals and reagents were of analytical grade and used without further purification. Zeolite L crystals (ZL) were prepared under hydrothermal conditions from a gel mixture using the procedure described by Sadegh Hassani et al. [62]. Scanning electron microscopy (SEM) allowed the determination of the ZL particle size to be 200 nm and the Si/Al ratio was equal to 4. To remove water and organic debris, the ZL was heated under vacuum at 250 °C.

### 4.2. Preparation of the Samples

In a first step, nine hydrogels, based on PVA and CS in different ratios and loaded with arginine and caffeine, were prepared by the freeze–thaw technique. A 5% solution of PVA was prepared by mixing PVA with double distilled water at 90 °C for 6 h. The bioactive component (arginine and/or caffeine 2%) was added to the aqueous PVA solution, and later the chitosan solution (2%) was added in various reports.

**Figure 6:**
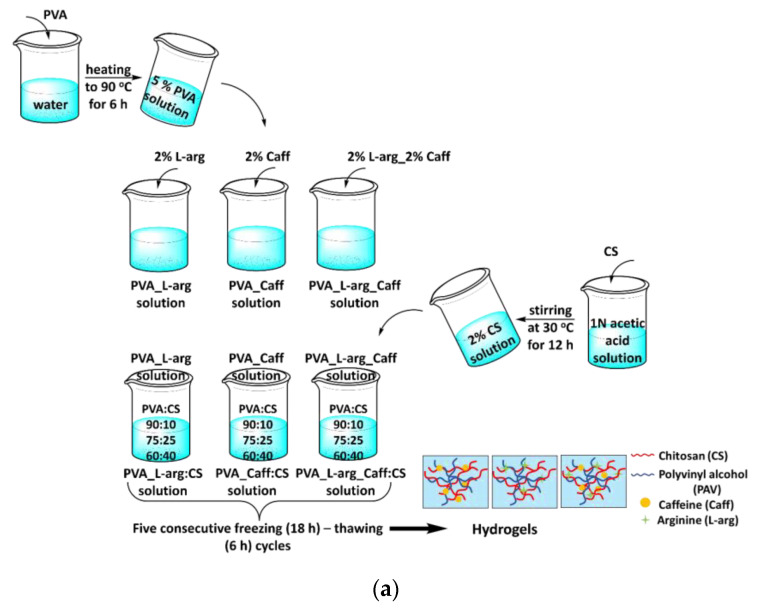

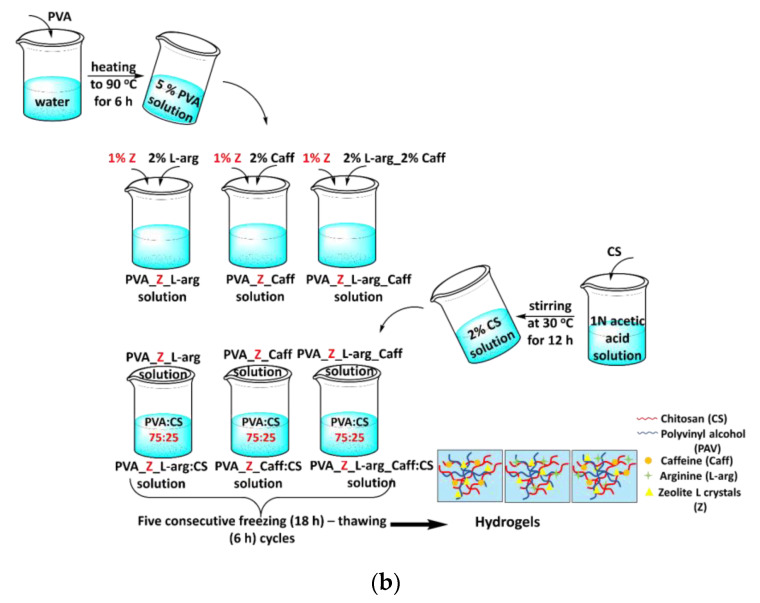
Experimental presentation of obtaining hydrogels: (**a**) without zeolite and (**b**) with zeolite.

A 2% CS solution was prepared by dissolving chitosan in 1N acetic acid solution by stirring at 30 °C for 12 h. Based on the preliminary results obtained from the nine hydrogels, the best APV:CS ratio was selected, and an inorganic material, zeolite (Z), was added in order to increase the release time of the active substance. All samples were subjected to five consecutive freezing (18 h)–thawing (6 h) cycles. During the freezing process, the temperature was maintained at −20 °C, and during the defrosting process it was +20 °C [63]. All the obtained hydrogels were freeze-dried over 48 h at −46 °C and stored in a fridge at 5 °C. The composition of the samples is shown in Table 4 and the experimental presentation of obtaining hydrogels is shown in Figure 6.

### 4.3. Methods

#### 4.3.1. Attenuated Total Reflection Fourier Transform IR (ATR-FTIR) Spectroscopy

The IR absorption spectra of the analyzed samples were recorded using a Bruker Vertex 70 spectrometer (Bruker Optics, Ettlingen, Germany) equipped with a ZnSe crystal ATR accessory in the scan range of 4000–600 cm^−1^ at a resolution of 4 cm^−1^ at room temperature.

#### 4.3.2. Morphological Characterization Via Scanning Electron Microscopy (SEM)

The morphological characterization of the hydrogels was carried out on an environmental scanning electron microscope (ESEM) type Quanta 200 operating at 30 kV with secondary and backscattering electrons in low vacuum mode coupled with dispersive X-ray spectroscopy (EDX) in order to perform the elemental analysis on the film surface.

#### 4.3.3. Dynamic Water Vapor Sorption Capacity

The hydrogels’ capacity to capture water vapor was determined by using the gravimetric analyzer IGAsorp (Hiden Analytical, Warrington (UK)). The system measurements were fully automated and controlled by a user-friendly software package running on Microsoft^®^ Windows^TM^.

In order to study the water vapor sorption properties of hydrogels, initially the vapor pressure was increased in 10% humidity steps, each having a pre-established equilibrium time between 10 and 20 min. The ultrasensitive microbalance, with which the device is equipped, measures the change in the weight of the samples as the humidity is modified. The cycle was ended by decreasing the vapor pressure in steps to obtain also the desorption isotherms. Before sorption measurements, the samples were dried at 25 °C in flowing nitrogen (250 mL/min) until their weight was in equilibrium at RH below 1%. The water content was calculated using the following equation:

(1) $$Water\;content\;\left(\%\right)=\left(\frac{W_{t}-W_{d}}{W_{d}}\right)\cdot 100$$

*W_t_*—weight of the swollen samples at time *t*;

*W_d_*—weight of the dry sample.

#### 4.3.4. Estimation of Drug Loading and Entrapment Efficiency

The quantitative determination of L-arginine and caffeine, solubilized in phosphate buffer pH 7.4, was carried out by a spectrophotometric method (SPECORD 210 PLUS-223F2042C). The calibration curve for L-arginine was conducted in the concentration range 5–35 µg/mL and for caffeine in the range 5–25 µg/mL.

For the estimation of drug loading and entrapment efficiency, after the preparation of hydrogels, three samples were taken from each hydrogel from different areas. Then, the samples were placed in phosphate-buffered solution pH 7.4 and kept for 30 min in a sound field. The obtained solutions were centrifuged, filtered and samples were taken from the supernatant and analyzed spectrophotometrically. The experimental concentration value of the loaded drug was calculated from the calibration curve relationship. Using relations (2) and (3), the loading capacity (*LC*) and entrapment efficiency (*EE*) of the hydrogels were calculated [36].

(2) $$LC\left(\%\right)=\left(\frac{M_{actual\left(caff/L.arg\right)\;}}{M_{sample}}\right)\cdot 100$$

*LC*—loading capacity of caffeine or L-arginine in hydrogels;

*M_actual_*_(*caff/L.arg*)_—actual amount of caffeine or L-arginine loaded in the hydrogel sample;

*M_sample_*—actual amount of hydrogel sample.

(3) $$EE\left(\%\right)=\left(\frac{M_{actual(caff/L.arg)}}{M_{theoretical\;caff/L.\;arg\;}}\right)\cdot 100$$

*EE*—entrapment efficiency, %;

*M_actual_*_(*caff/L.arg*)_—actual amount of caffeine or L-arginine loaded in the hydrogel;

*M_theoretical caff/L.arg_*—theoretical amount of caffeine or L-arginine added in the hydrogel preparation.

#### 4.3.5. In Vitro Drug Release and Permeation Studies

To perform the in vitro diffusion study, Franz vertical diffusion cells of 1.5 cm internal diameter were used (Orchid Scientific Ltd., Ambad, India). The assays were performed on freshly slaughtered chicken skin that was purchased from a local slaughterhouse. In order to obtain this biological membrane, skin was excised from chicken legs from areas without pores, followed by the removal of fatty tissue. These membranes were cut in areas of 5 cm^2^ and placed in 10% glycerin before being mounted between the donor and receptor compartments. The temperature was maintained at 36.5 ± 0.5 °C by connecting the diffusion cells to a thermostatic water bath and the homogeneity of receptor fluid was maintained by magnetic stirring [64]. Individually samples of hydrogels containing the drugs (100 mg Caff and 125 mg L-arg) were placed in the donor compartment, directly on the biological membrane. Each receptor compartment was filled with 12 mL of isotonic phosphate buffer pH 7.4 solution [65] and the rotation was set at 100 rpm. The samples (0.5 mL aliquots) were withdrawn from the receptor cell at a regular time interval for a period of 4 h and replaced with the same volume of fresh medium. The amount of the drug released and passed through the membrane was analyzed spectrophotometrically with the methods described. The in vitro release profile and permeation expressed as the cumulative amount of Caff and L-arg transported across the membrane per cm^2^ vs. incubation time was plotted in GraphPad^®^.

#### 4.3.6. Analysis of In Vitro Drugs Release Kinetics

To study the mechanism in which L-arg and Caff leave the polymeric matrix, the in vitro release profiles were correlated with various kinetic models. The mathematical models applied were for zero-order kinetics (the ideal case, constant release of the drug from the polymer matrix), first-order kinetics (the release rate of the drug depends on its concentration), the Higuchi model (the release of the drug is carried out by diffusion), and the Korsmeyer–Peppas model (the value of n, i.e., the release exponent, indicates the mechanism of drug release from a matrix) [59,66].

The data fitting was carried out by linear or non-linear regression using Matlab 7.1. Akaike information criterion (AIC) and the correlation coefficient R^2^ were the criteria for selecting the model that most faithfully depicted the release profile of each studied formulation. A prediction of the model that is as good as possible requires R^2^ to be as close to 1 as possible, and the AIC to have minimum values [67,68].

## Figures and Tables

**Table 1:** Hydrogel loading capacity (LC) and entrapment efficiency.

Sample	PVA:CS %	L-arg %	Caff %	*LC_L-arg_* ± SD %	*LC_Caff_* ± SD %	*EE_L-arg_* ± SD %	*EE_Caff_* ± SD %
PVA_L-arg:CS	90:10	2	-	1.98 ± 0.0002	-	99.05 ± 0.2	-
	75:25	2	-	1.98 ± 0.0003	-	99.13 ± 0.5	-
	60:40	2	-	1.99± 0.0002	-	99.05 ± 0.7	-
PVA_Caff:CS	90:10	-	2	-	1.97 ± 0.0003	-	98.84 ± 0.5
	75:25	-	2	-	1.97 ± 0.0003	-	98.80 ± 0.6
	60:40	-	2	-	1.98 ± 0.0002	-	99.16 ± 0.3
PVA_L-arg_Caff:CS	90:10	2	2	-	1.99 ± 0.0002	-	99.16 ± 0.3
	75:25	2	2	-	1.97 ± 0.0002	-	98.89 ± 0.3
	60:40	2	2	-	1.99 ± 0.0001	-	99.27 ± 0.1
PVA_Z_L-arg:CS	75:25	2	-	1.92 ± 0.001	-	95.88 ± 0.46	-
PVA_Z_Caff:CS	75:25	-	2	-	1.84 ± 0.003	-	91.80 ± 1.03
PVA_Z_L-arg_Caff:CS	75:25	2	2	-	1.70 ± 0.003	-	85.09 ± 1.02

**Table 2:** Data fitting results of in vitro L-arg release profile from hydrogels.

Kinetic Model	Model Coefficients *	Sample			
		PVA_L-arg:CS 90:10	PVA_L-arg:CS 75:25	PVA_L-arg:CS 60:40	PVA_Z_L-arg:CS 75:25
Zero-order	K_0_ (µg/h)	34.6604	33.4181	33.0437	30.0594
	R^2^	0.7397	0.8173	0.7749	0.9400
	AIC	90.4906	86.1915	89.5444	68.7646
First-order	K_1_ (h^−1^)	1.5081	1.1985	1.4667	0.7091
	R^2^	0.9540	0.9434	0.9098	0.9893
	AIC	52.0653	54.4257	59.4848	34.4222
Higuchi	K_H_ (h^−0.5^)	68.4502	58.6092	60.0338	50.3189
	R^2^	0.9207	0.9626	0.9395	0.9935
	AIC	67.5030	58.0864	66.1111	36.3008
Korsmeyer–Peppas	n	0.31	0.35	0.30	0.54
	K_P_ (h^−n^)	71.1471	65.8352	70.2094	48.8744
	R^2^	0.9728	0.9890	0.9898	0.9937
	AIC	47.2008	35.1684	33.1479	30.8432

* K_0_ = constant of zero-order release rate, K_1_ = constant of first-order release rate, K_H_ = constant of Higuchi model release rate, K_P_ = constant of Korsmeyer–Peppas model release rate.

**Table 3:** Data fitting results of in vitro Caff release profile from hydrogels.

Kinetic Model	Model Coefficients *	Sample							
		PVA_Caff:CS 90:10	PVA_Caff:CS 75:25	PVA_Caff:CS 60:40	PVA_L-arg_Caff:CS 90:10	PVA_L-arg_Caff:CS 75:25	PVA_L-arg_Caff:CS 60:40	PVA_Z_Caff:CS 75:25	PVA_Z_L-arg_Caff:CS 75:25
Zero-order	K_0_ (µg/h)	31.7189	33.2027	32.3685	29.8304	30.4852	31.0108	29.0580	26.4436
	R^2^	0.8904	0.8328	0.8716	0.9540	0.9480	0.9445	0.9688	0.9769
	AIC	81.7390	86.3340	83.4886	69.4658	70.5143	68.7899	61.7122	49.9559
First-order	K_1_ (h^−1^)	0.9182	1.1136	1.0039	0.6955	0.7318	0.7357	0.6162	0.4852
	R^2^	0.8788	0.8905	0.8873	0.9630	0.9658	0.9754	0.9732	0.9695
	AIC	62.8648	61.9319	62.1767	49.2599	48.8112	46.1660	46.1221	47.3676
Higuchi	K_H_ (h^−0.5^)	54.6274	58.0423	56.1032	49.4901	50.9705	51.2613	48.1464	43.0442
	R^2^	0.9803	0.9651	0.9772	0.9939	0.9924	0.9855	0.9824	0.9609
	AIC	51.2550	59.7086	54.0957	28.2244	30.6220	43.9314	47.4270	59.1188
Korsmeyer–Peppas	n	0.38	0.33	0.36	0.54	0.55	0.58	0.64	0.78
	K_P_ (h^−n^)	60.0406	66.2640	62.3882	48.3429	48.9719	49.0189	42.6281	33.3417
	R^2^	0.9878	0.9915	0.9903	0.9953	0.9933	0.9878	0.9925	0.9838
	AIC	35.0171	30.6338	32.2345	24.5002	29.6927	39.0642	31.5291	41.1629

* K_0_ = constant of zero-order release rate, K_1_ = constant of first-order release rate, K_H_ = constant of Higuchi model release rate, K_P_ = constant of Korsmeyer–Peppas model release rate.

**Table 4:** Composition of the hydrogels.

Sample	PVA (wt%)	CS (wt%)	L-arg (wt%)	Caff (wt%)	L-arg–Caff (wt%)	Z (wt%)
PVA_L-arg:CS 90:10	90	10	2	-	-	-
PVA_L-arg:CS 75:25	75	25	2	-	-	-
PVA_L-arg:CS 60:40	60	40	2	-	-	-
PVA_Caff:CS 90:10	90	10	-	2	-	-
PVA_Caff:CS 75:25	75	25	-	2	-	-
PVA_Caff:CS 60:40	60	40	-	2	-	-
PVA_L-arg_Caff:CS 90:10	90	10	-	-	2	-
PVA_L-arg_Caff:CS 75:25	75	25	-	-	2	-
PVA_L-arg_Caff:CS 60:40	60	40	-	-	2	-
PVA_Z_L-arg:CS 75:25	75	25	2	-	-	1
PVA_Z_Caff:CS 75:25	75	25	-	2	-	1
PVA_Z_L-arg_Caff:CS 75:25	75	25	-	-	2	1

## Data Availability

Not applicable.
